# Prognostic value of the albumin-to-alkaline phosphatase ratio on urologic outcomes in patients with non-metastatic renal cell carcinoma following curative nephrectomy

**DOI:** 10.7150/jca.34029

**Published:** 2019-08-29

**Authors:** Aidan Xia, Yuming Chen, Jingfeng Chen, Yue Pan, Lianmin Bao, Xiaomin Gao

**Affiliations:** 1 Department of Hematology, The Third Clinical Institute Affiliated to Wenzhou Medical University, People's Hospital of Wenzhou, Wenzhou, Zhejiang province, 325006, P.R. China; 2 Department of Urology, Affiliated Hospital of Yangzhou University, Yangzhou, Jiangsu province, 225001, P.R. China; 3 Department of Anorectal surgery, sixth affiliated hospital of Wenzhou medical university (Lishui People's Hospital), Lishui, Zhejiang province, 323000, P.R. China; 4 Department of Urology, The First Affiliated Hospital of Wenzhou Medical University, Wenzhou, Zhejiang province,325006, P.R. China; 5 Department of Respiratory, Rui'an People's Hospital, The Third Affiliated Hospital of the Wenzhou Medical University, Wenzhou, Zhejiang province, 325200, P.R. China; 6 Department of Urology, Changhai Hospital, Second Military Medical University, Shanghai, 200433, P.R. China

**Keywords:** renal cell carcinoma, albumin-to-alkaline phosphatase ratio, prognosis, serum biomarker

## Abstract

**Background**: Few studies focused on the relationship between the albumin-to-alkaline phosphatase ratio (AAPR) and the urologic outcomes in patients with non-metastatic renal cell carcinoma (RCC) following curative surgery. The aim of this study was to evaluate the prognostic value of preoperative AAPR in non-metastatic RCC patients.

**Methods**: The prognostic value of AAPR was evaluated in a primary cohort with 419 non-metastatic RCC patients following curative radical or partial nephrectomy and then further validated in an independent cohort consisting of 204 patients. A nomogram was developed based on the independent predictors, and its predictive value was assessed.

**Results**: Kaplan-Meier survival analysis demonstrated that patients with low AAPR levels were significantly associated with worse overall survival (OS) and cancer-specific survival (CSS) compared with patients with high AAPR levels both in two cohorts. Univariate and multivariate analyses revealed that low AAPR was an independent risk factor for OS (HR = 2.745; 95%CI, 1.266-5.953; *P* = 0.011) and CSS (HR = 3.042; 95%CI, 1.278-7.243; *P* = 0.012). Moreover, subgroup analysis (Fuhrman grade G1+G2 and Fuhrman grade G3+G4; T1+T2 stage and T3+T4 stage) revealed that low AAPR was also related to worse urological outcomes. Although no significant differences between patients with low AAPR and patients with high AAPR can be observed with regard to CSS under Fuhrman grade G1+G2 (*P*=0.058) and T1+T2 stage (*P*=0.318), there was a worse CSS trend in low AAPR patients. The established nomograms for OS and CSS were well calibrated and had moderate discriminative ability (concordance index: 0.821 and 0.839, respectively)

**Conclusions**: Preoperative AAPR might be an independent prognostic factor in patients with non-metastatic RCC. The ratio should be applied in RCC patients for risk stratification and clinical decision-making.

## Introduction

Renal cell carcinoma (RCC) is one of the most common cancers in urology, accounting for more than 90% of all kidney cancers 1-3. The global incidence of RCC has increased by approximately 2% during the last two decades 2. There were roughly 27,400 new cases and 117,000 kidney-cancer related deaths worldwide in 2008 4. In Asia, the number of cases of kidney cancer per 100,000 person years in Japan, Singapore, Hong Kong, Shanghai, China, and India were 6.5, 4.3, 3.8, 2.9, and 2.0, respectively, with Japan having the highest incidence 5. At present, surgical resection remains the most effective therapy for clinically localized disease 2. Nevertheless, there remain approximately 30% of patients who will inevitably suffer from local or distant recurrence following curative nephrectomy 2. Therefore, an increasing number of researchers have paid attention to seeking useful pre- and post-operative predictors to categorize patients with worse outcomes at early time-points.

Several prognostic models have been developed and have been well-confirmed in external validation cohorts 6-9. However, the prognostic value of these models can be potentially improved because none of them has been applied in clinical practice because of the time-consuming and expensive nature of assays, lack of standardization, and non-reproducibility 10. Therefore, a new potential prognostic factor should be developed that is cheap and easily detected in a standardized manner. At the moment, some biomarkers from blood have been established, including the AST/ALT (De Ritis) ratio, the neutrophil-to-lymphocyte ratio, and the prognostic nutritional index 10-12, although their results remain inconsistent and controversial. Recently, the albumin-to-alkaline phosphate ratio (AAPR), a novel prognostic factor, has been demonstrated to be significantly associated with poorer urologic outcomes for hepatocellular carcinoma (HCC), metastatic nasopharyngeal carcinoma, and upper tract urothelial carcinoma (UTUC) 13-17. However, it has not yet been studied in non-metastatic RCC patients following curative nephrectomy. Therefore, the aim of this study was to evaluate the potential prognostic impact of preoperative AAPR in patients with non-metastatic disease.

## Materials and Methods

**Figure 1:**
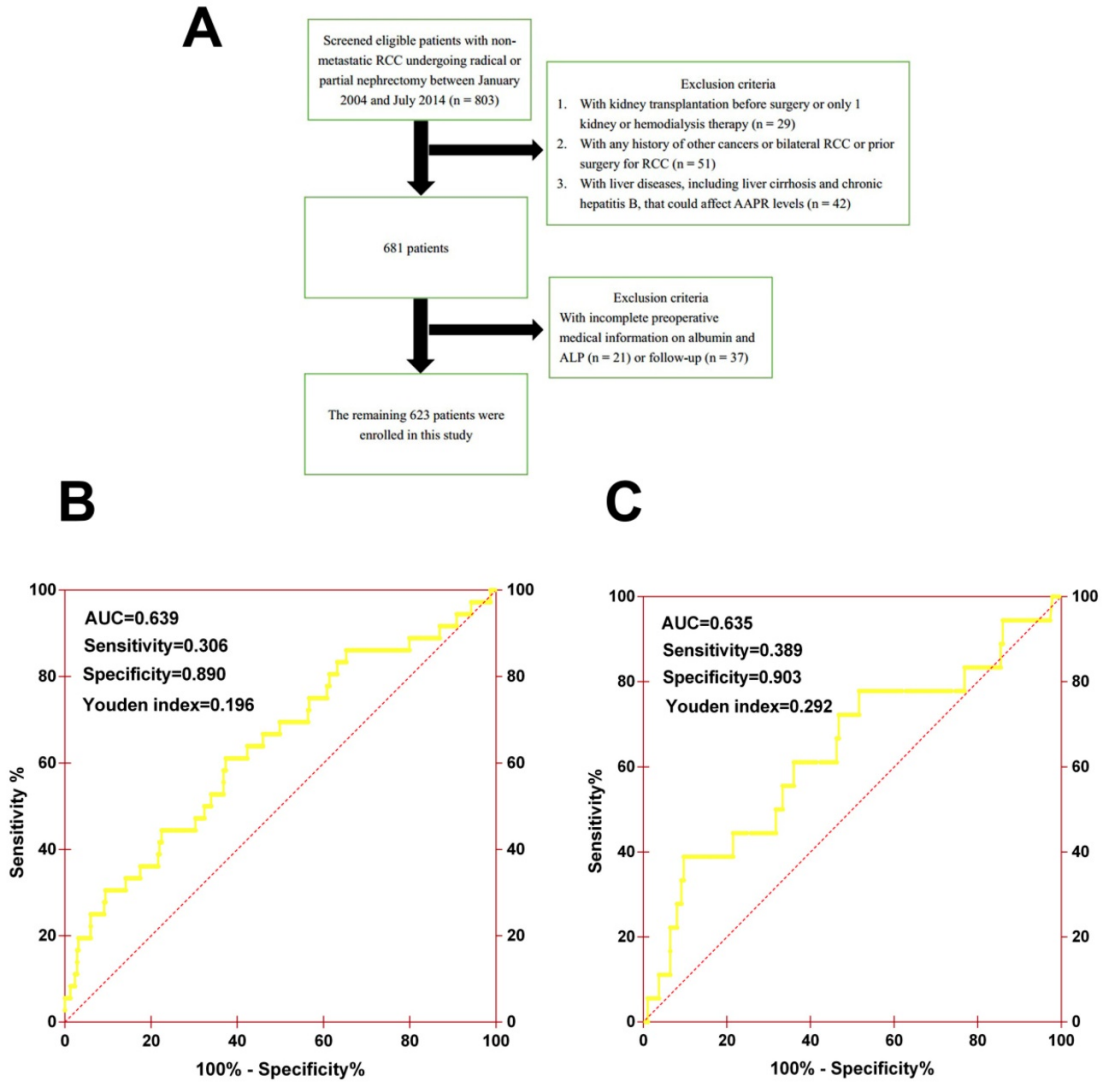
(A) The patient selection flowchart. (B) The ROC curve of AAPR for OS in primary cohort. (C) The ROC curve of AAPR for OS in validation cohort.

**Patients.** This retrospective study included 803 consecutive patients diagnosed with non-metastatic RCC (pathological T1-4N0M0) between January 2004 and July 2014 at the Urologic Department of The First Affiliated Hospital of Wenzhou Medical University, China. All underwent curative radical or partial nephrectomy and none received neo-adjuvant chemotherapy before surgery. The exclusion criteria were patients who: (1) underwent kidney transplantation before surgery or had only 1 kidney or hemodialysis therapy (n = 29); (2) had any history of other cancers or bilateral RCC or prior surgery for RCC (n = 51); (3) had liver diseases, including cirrhosis and chronic hepatitis B, that could affect AAPR levels (n = 42); and (4) had incomplete preoperative medical information on albumin and ALP (n = 21) or follow-up (n = 37) (Figure 1A). Finally, a total of 623 patients were enrolled in this study, among which 419 patients were randomly assigned to the primary cohort and 204 patients were assigned to the validation cohort. This study was approved by the ethics committee of The First Affiliated Hospital of Wenzhou Medical University. The informed consent was not required for this study.

**Methods.** Clinicopathological records and data on laboratory assessments, including albumin (ALB) and alkaline phosphatase (ALP), were collected and retrospectively analyzed. The cutoff value of AAPR was determined by performing an ROC analysis for evaluating OS, and 0.39 was chosen as the final cutoff value because it had the maximum Youden index value (sensitivity: 30.6%; specificity: 89.0%; Youden index: 0.196) (Figure 1B). Patients were then generally followed up every 3 to 6 months for the first 2 years and annually after surgery for blood and urine tests, cystoscopy, and image examination. Information on death was obtained from outpatient medical records, telephone interviews, or the patient's social security death index.

**Statistical analysis.** Statistical analyses were performed using the SPSS software package version 25.0 (IBM, Armonk, NY), and two-sided *P* value < 0.05 were considered significant. The Pearson chi-square test was used to evaluate the association of clinicopathologic characteristics with AAPR. The overall survival (OS) rates and cancer-specific survival (CSS) rates were estimated using the Kaplan-Meier method in primary cohort and validated cohort. To determine the independent prognostic factors, univariate analysis and multivariate Cox regression analysis were performed. Variables with *P* < 0.05 in the univariate analysis were included in the subsequent multivariate model. Nomograms for probability of OS and CSS were established based on the results of the multivariate analysis using the R software (Version 3.6.0) with the packages rms, Hmisc, and ggplots. Calibration plot, concordance index (c-index), and ROC analysis were applied to evaluate the performance of nomograms.

## Results

### Patient characteristics

The patients' clinicopathological characteristics of both two cohorts are summarized in Table 1. In the primary cohort, there were 266 (63.5%) males and 153 (36.5%) females. The mean age was 61.0±12.9 years and 173 (41.3%) patients were 65 years of age or older. The median follow-up duration was 50.0 (30.4 - 83.0) months. During follow up, a total of 36 (8.6%) patients died, among which 27 (6.4%) patients died of cancer-specific causes. In the validation cohort, there were 127(62.3%) males and 84 (41.2%) patients were 65 years of age or older. The mean age was 62.4±11.7 years. The median follow-up duration was 50.2 (29.8 - 83.1) months. During follow up, a total of 18 (8.8%) patients died, among which 10 (4.9%) patients died of cancer-specific causes.

### Associations between AAPR and clinicopathological characteristics of the primary cohort

The clinicopathological characteristics of the cohort according to the preoperative values of AAPR are shown in Table 2. The median value of AAPR was 0.58 (0.46-0.71). Higher serum ALT (*P*=0.004) and AST (*P*=0.016) levels and anemia (*P*=0.001) can be more commonly observed in patients with low AAPR. Additionally, low AAPR patients were older (*P*=0.004) than high AAPR patients.

### Prognostic significance of AAPR

**Figure 2:**
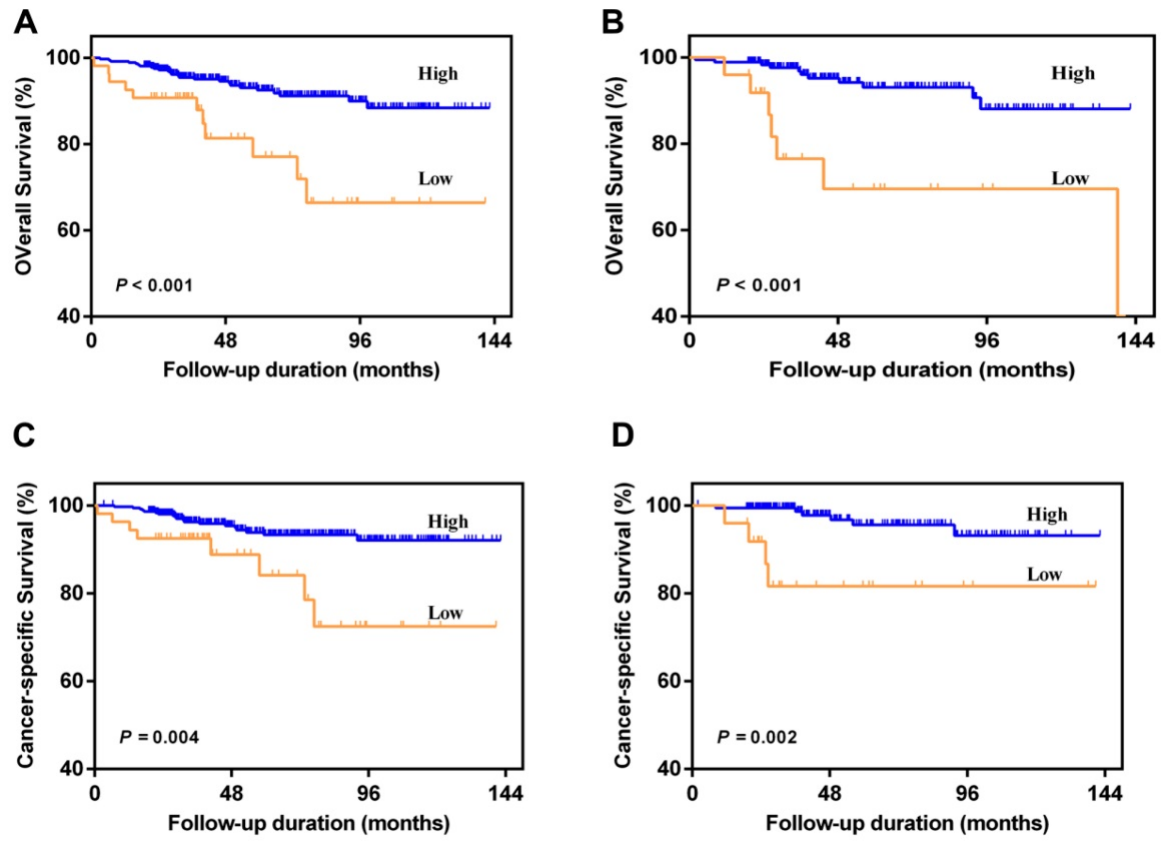
Kaplan-Meier survival curves for OS and CSS of patients with non-metastatic RCC according to AAPR levels. Patients with low AAPR were associated with worse OS and CSS in primary cohort (A and C) and validation cohort (B and D).

Patients with AAPR < 0.39 were significantly associated with worse OS and CSS compared with patients with AAPR ≥ 0.39 in primary cohort (Figure 2A and 2B) and validation cohort (Figure 2C and 2D). In primary cohort, the 5-year OS and CSS rates were 92.5% and 93.3% in patients with AAPR ≥ 0.39, and 77.1% and 84.1% in patients with AAPR < 0.39, respectively. In validation cohort, the 5-year OS and CSS rates were 93.1% and 95.6% in patients with AAPR ≥ 0.39, and 69.6% and 81.6% in patients with AAPR < 0.39, respectively.

In the univariate analysis, AAPR < 0.39 was significantly associated with poorer OS and CSS. Variables with *P* < 0.05 in the univariate analysis were included in the subsequent multivariate analysis and the results revealed that AAPR < 0.39 was identified as an independent risk factor of OS (HR = 2.745; 95%CI, 1.266-5.953; *P* = 0.011) and CSS (HR = 3.042; 95%CI, 1.278-7.243; *P* = 0.012) (Table 3). Other variables were also determined as independent predictors, including old age, higher pathologic T stage, and tumor necrosis.

**Figure 3:**
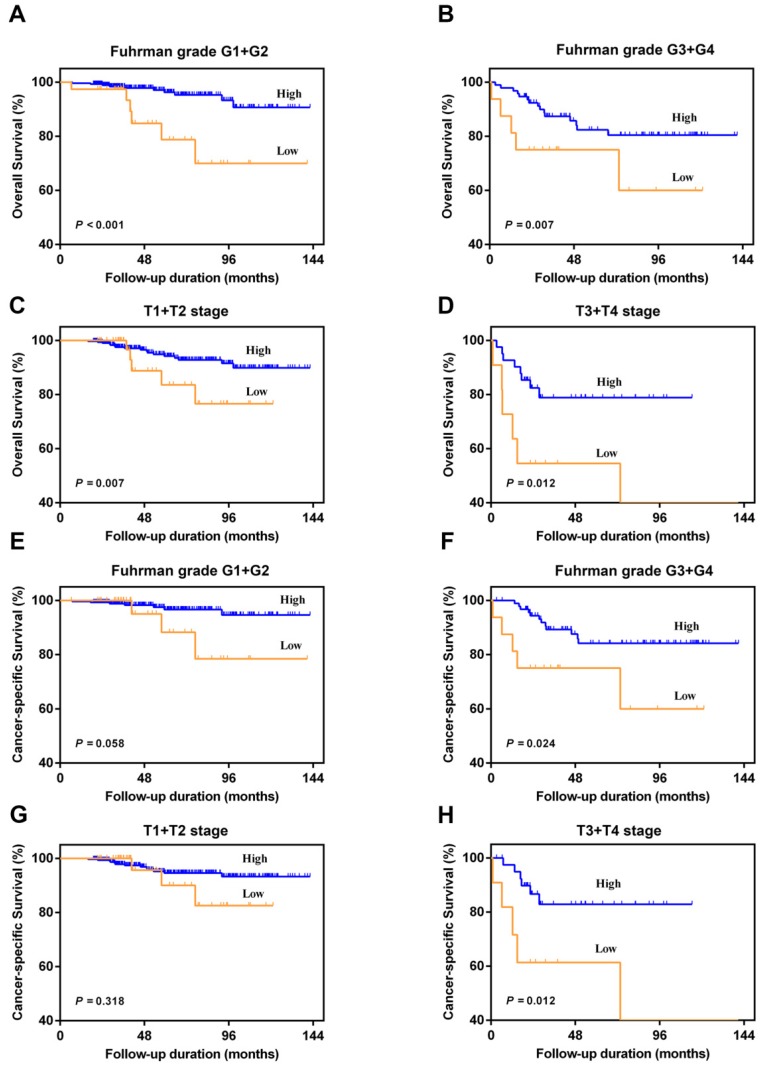
Kaplan-Meier curves for OS and CSS according to AAPR in the Fuhrman grade G1+G2 (A and E), Fuhrman grade G3+G4 (B and F), pathological T1+T2 stage (C and G), and pathological T3+T4 stage (D and H).

In the subgroup analysis, we divided patients into Fuhrman grade G1+G2 group and Fuhrman grade G3+G4 group, or T1+T2 stage group and T3+T4 stage group. The results showed that AAPR < 0.39 was also significantly associated with poorer urologic outcomes among four different subgroups (*P* < 0.05) (Figure 3). Although no significant differences between patients with AAPR < 0.39 and patients with AAPR ≥0.39 can be observed with regard to CSS under Fuhrman grade G1+G2 (*P*=0.058) and T1+T2 stage (*P*=0.318) (Figure 3E and 3G), there was a worse CSS trend in low AAPR patients.

### The nomogram and its performance

**Figure 4:**
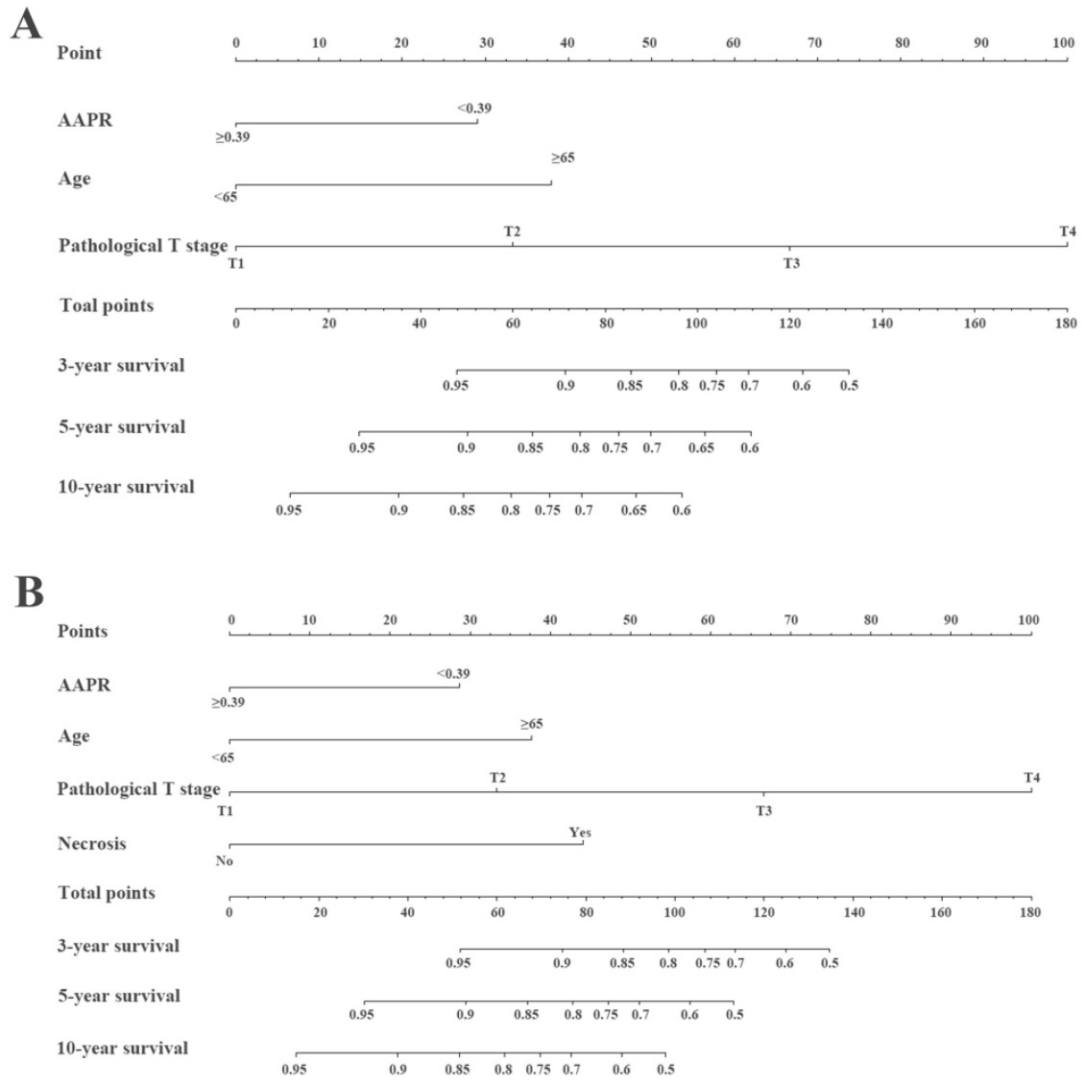
Construction of preoperative nomogram with AAPR and other significant factors that predicted the probability of non-metastatic RCC for OS (A) and CSS (B). To use the nomogram, the value of individual patients with RCC is located on each variable axis, and a line is depicted upward to determine the number of points received for each variable value. Subsequently, the sum of these numbers is located on the Total Point axis, and a line is drawn downward to the survival axes to determine the likelihood of 3-, 5-, and 10-year survival of OS or CSS.

**Figure 5:**
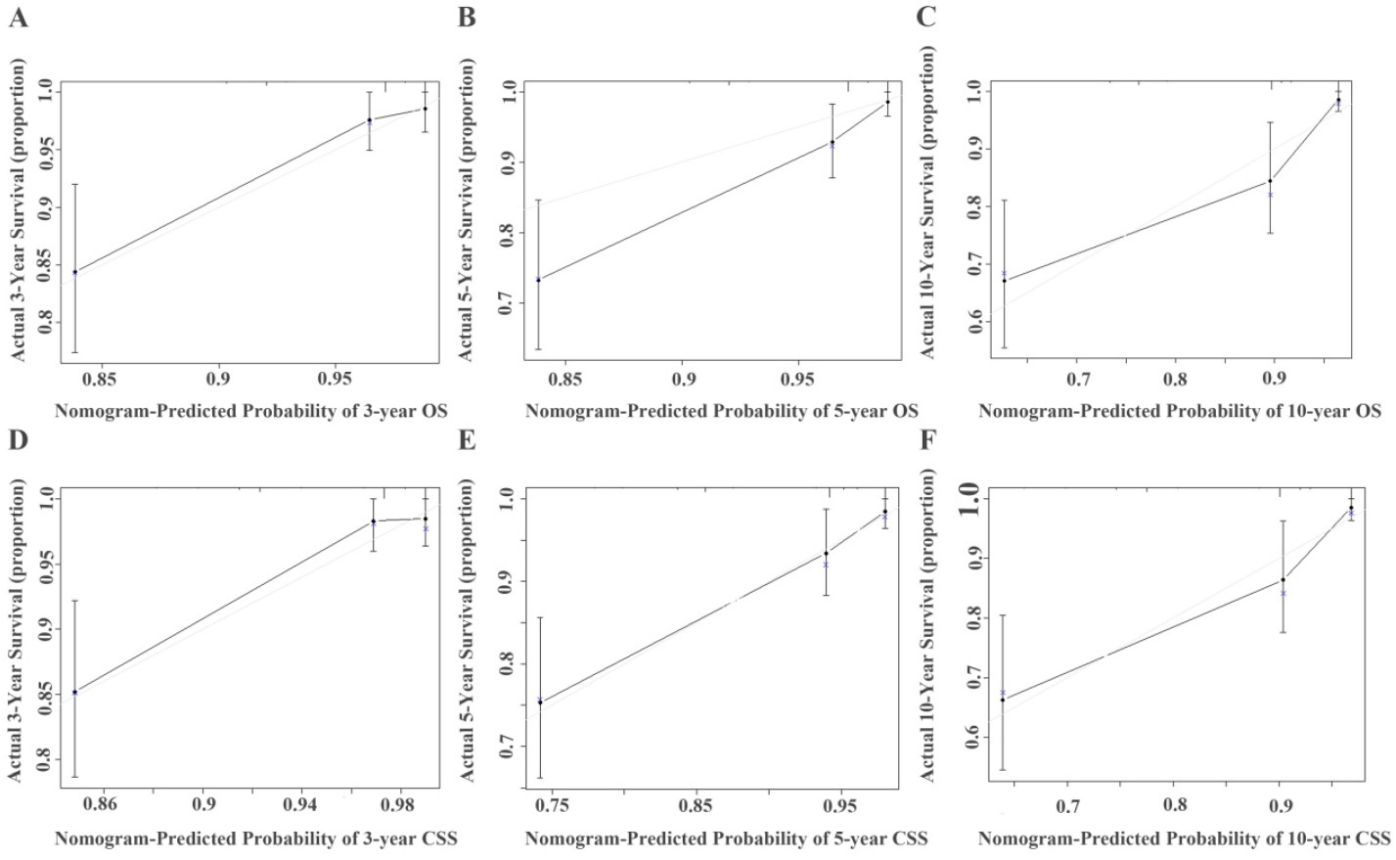
Calibration curve for predicting 3-, 5-, and 10-year survival of OS (A, B, and C) or CSS (D, E, and F) in non-metastatic RCC patients. The actual OS or CSS rates are plotted on the y-axis and nomogram-predicted OS or CSS rates are plotted on the x-axis.

**Figure 6:**
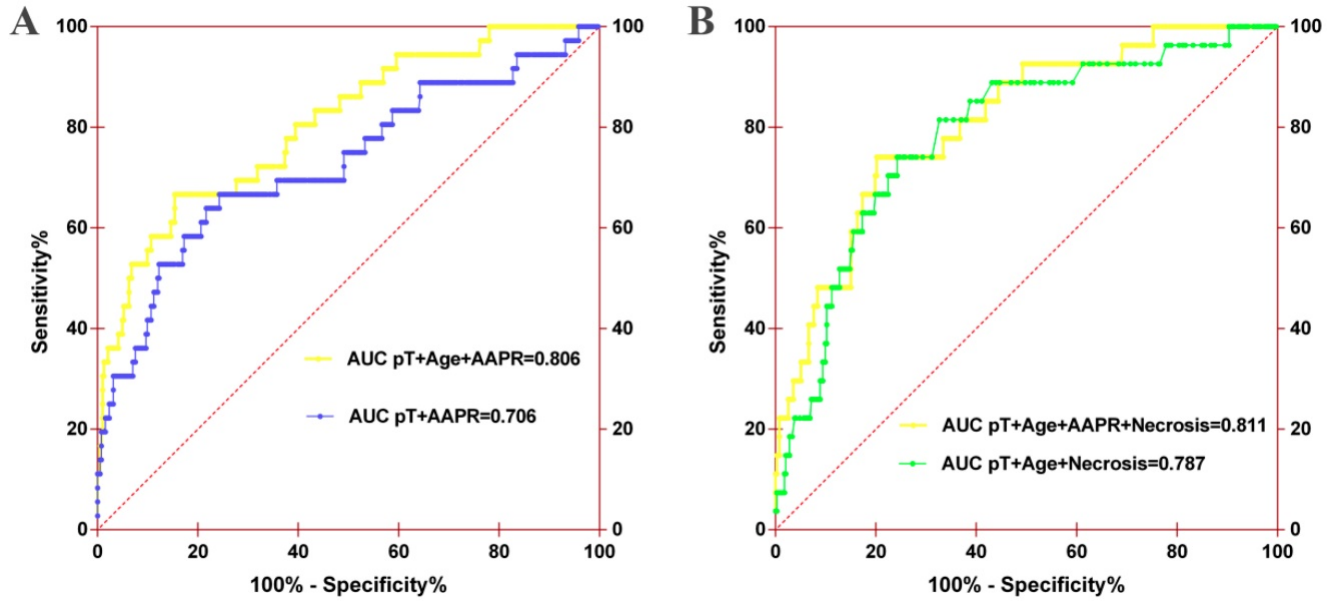
ROC analysis of the nomogram model for OS (A) or CSS (B). For OS, the AUC of the nomogram model was 0.706 when assessed by pT and age, which increased to 0.806 when the AAPR was added. For CSS, the AUC of the nomogram model increased from 0.787 to 0.811 when AAPR was added.

The prognostic nomograms for OS (Figure 4A) and CSS (Figure 4B) were depicted by independent indicators in the multivariate analysis. Each predictor in the nomogram was assigned a score (top scale). Thereafter, the sum of these scores implied the probability of 3-, 5-, and 10-year OS or CSS (bottom scale). The c-indexes for the nomogram of OS and CSS were 0.821 (95%CI, 0.750-0.892) and 0.839 (95%CI, 0.7557-0.922), respectively (Table 4), indicating moderate discriminative ability of these two models (low discriminative ability: 0.50-0.70; moderate discriminative ability: 0.71-0.90; high discriminative ability: 0.90-1). The calibration plots of the nomograms were developed (Figure 5), which demonstrated that the nomograms were well-calibrated. As shown in Figure 6 and Table 5, the AUC of the nomogram for OS was 0.806 (95% CI, 0.728-0.884), with the sensitivity, specificity, and Youden index of 66.67%, 84.60%, and 0.513, respectively. The AUC of the nomogram for CSS was 0.811 (95% CI, 0.729-0.892), with the sensitivity, specificity, and Youden index of 74.07%, 79.85%, and 0.539, respectively.

### Evaluation of the predictive ability of AAPR for OS and CSS

By incorporating AAPR into the developed models, the c-indexes of the nomograms for OS and CSS increased from 0.772 (95% CI, 0.690-0.854) to 0.821 (95% CI, 0.750-0.892), and from 0.809 (95% CI, 0.723-0.895) to 0.839 (95%CI, 0.756-0.922), respectively (Table 4).

Furthermore, AUC comparison demonstrated that the AUC of the nomogram for OS increased from 0.706 (95% CI, 0.601-0.812) to 0.806 (95% CI, 0.728-0.884) when AAPR was incorporated into the model (Figure 6A). The AUC of the nomogram for CSS also improved from 0.787 (95% CI, 0.698-0.875) to 0.811 (95% CI, 0.729-0.892) when AAPR was added (Figure 6B). Other predictive parameters of nomograms for OS and CSS also were improved (Table 5). These findings revealed that AAPR could be a useful indicator of urologic outcomes in patients with non-metastatic RCC, which should be applied in patients with RCC for risk stratification and clinical decision-making.

## Discussion

Various risk assessment models have been previously developed to predict the prognosis in patients with or without RCC patients after surgery, including the Leibovich prognosis score 7, Mayo Clinic SSIGN (stage, size, grade, and necrosis) 18, and UISS (UCLA Integrated Staging System) 19. Several studies have identified several immunohistochemical biomarkers, genomic approaches, and nomograms as significantly independent factors for postoperative survival in RCC patients 20, 21. In the present study, AAPR, a novel risk factor, was introduced and its prognostic value was evaluated and validated in this study. The cutoff value of AAPR was determined to be 0.39 by performing ROC analysis, and AAPR < 0.39 was found to be statistically correlated with old age, anemia, and higher pathological T stage. Subsequently, AAPR < 0.39 was revealed to be associated with poorer OS and CSS in primary cohort and confirmed in validation cohort. Furthermore, decreased AAPR was identified as an independent risk predictor for OS and CSS in non-RCC patients after surgery according to multivariate analysis. The predictive abilities of the developed nomograms also increased when AAPR were incorporated into these models.

AAPR was first reported by Anthony et al. in 2015. They found that AAPR was an independent prognostic indicator for hepatocellular carcinoma (HCC) with the highest c-index and χ^2^ among other liver biochemical parameters 15. Researchers then demonstrated that AAPR was also an independent predictor of advanced HCC and metastatic nasopharyngeal carcinoma, and its predictive ability was significantly better than that of ALB or ALP alone 13, 14. Recently, Tan et al. reported that AAPR was significantly associated with worse survival outcomes in UTUC with relative high AUC 17. With respect to its superior predictive accuracy for cancer outcomes and absence of studies evaluating the prognostic role in RCC patients, we performed this study and found the same results as mentioned previously.

It remains unclear why lower AAPR increases the risk of tumor relapses and mortality; however there is one possibility that should be addressed: AAPR is calculated from serum ALB concentration divided by serum ALP concentration, indicating that nutritional deficiency and systemic inflammatory response might be involved in the development and progress of RCC when AAPR performed for its prognostic impact on tumor recurrence and metastasis. ALB is specifically synthesized by liver, where it is not only an important nutritional index, but also it is associated with systemic immunological response to inflammatory or tumor 22. ALB has the ability to stabilize cell growth and proliferation, modulate immune reactions, and exert antioxidant effects against carcinogens 23. As a result, the presence of low ALB or hypoalbuminemia may lead to impairment of immunity and poor anti-cancer responses 24. Previous studies have reported that ALB was a reliable predictive tool in various cancers, including HCC, RCC, and prostate cancer 25-27. ALP is a hydrolase enzyme that is primarily located in the kidney, liver, and bone. Serum ALP levels commonly increase in patients with HCC, kidney disease, and bone metastasis 13. Furthermore, ALP was also identified as independent risk factor in various cancers, including HCC, nasopharyngeal carcinoma, and RCC 28-30.

In the present study, the optimal cutoff value was obtained from the maximal Youden index value by performing ROC analysis. This cutoff value, 0.39 for AAPR, was suggested as a superior prognostic level according to HR. Nevertheless, the cutoff-point was not consistent with those from previous studies 15-17, 27 and was close to that of one study 14. This common problem can also be observed in studies of other prognostic biomarkers, including the platelet-lymphocyte ratio and neutrophil-lymphocyte ratio 31. Several points are needed to be taken into account: the application in various cancers, cohorts with varying sample sizes, follow-up periods, survival end-points, and assay methods for AAPR, as well as absence of standardized methods to determine optimal cutoff value. Furthermore, although AAPR was still identified as an independent predictor, the proportion of patients with lower AAPR and pathologic T3 and T4 stage in the primary cohort of our study were only 54 (12.9%) and 54 (12.2%), relatively lower than those of other studies. For example, Tan et. al. retrospectively assessed 692 patients with UTUC after surgery in 2003 and 2016. The cutoff value of AAPR was determined to be 0.58 by performing ROC analysis, and they found that lower AAPR was significantly associated with worse prognosis in UTUC patients. However, there were 443 (64.0%) patients with AAPR < 0.58, and 342 (49.4%) patients with pathologic T3 and T4. Therefore, further studies are needed to identify the best cutoff value according to particular cancers.

There were some limitations to this study. First, our study was retrospectively designed in a single institution, possibly giving rise to selection bias. Nevertheless, our department is the largest urologic center with the largest sample size for patients with RCC in the south of Zhejiang Province; therefore, our data were representative and reliable. Second, we were unable to include C-reactive protein due to deficiency in some patients. We included preoperative hemoglobin instead because it is a similar predictor of urologic outcomes. Third, the optimal cutoff for AAPR also requires prospective validation. Fourth, the effects of dynamic changes in AAPR on long-term survival remain to be evaluated. Last, the prognostic value and mechanisms of AAPR are required to be evaluated in further prospective studies and basic researches. Fifth, our results revealed that low AAPR was also related to worse urological outcomes in subgroup analysis; however, Tan et al. found no relationship between AAPR and urologic outcomes in low-grade UTUC patients 17. Therefore, the impact of AAPR on urologic outcomes in different subgroups remains to be investigated.

## Conclusion

To the best of our knowledge, this was first study to assess the prognostic impact of AAPR in non-metastatic RCC patients following curative surgery. AAPR< 0.39 was an independent predictor of worse OS and CSS in RCC patients. Prospective studies and investigation of potential mechanisms regarding the close correlation between lower AAPR and inferior survival outcomes in non-metastatic RCC are required.

## Figures and Tables

**Table 1:** Characteristics of primary and validation cohorts

Variables	Primary cohort (n=419)	Validation cohort (n=204)
Age, years		
>65	173(41.3%)	84(41.2%)
≤65	246(58.7%)	120(58.8%)
Gender		
Male	266(63.5%)	127(62.3%)
Female	153(36.5%)	77(37.7%)
ASA grade		
≥3	23(5.5%)	17(8.3%)
<3	396(94.5%)	187(91.7%)
BMI, kg/m^2^		
≥25	92(22.0%)	57(27.9%)
<25	327(78.0%)	147(72.1%)
DM		
Yes	143(34.1%)	67(32.8%)
No	276(65.9%)	137(67.2%)
Hypertension		
Yes	173(41.3%)	85(41.7%)
No	246(58.7%)	119(58.3%)
Anemia		
Yes	65(15.5%)	25(12.3%)
No	354(84.5%)	179(87.7%)
Surgical approach		
Partial nephrectomy	87(20.8%)	45(22.1%)
Radical nephrectomy	332(79.2%)	159(77.9%)
CKD stage		
CKD 1	292(69.7%)	134(65.7%)
CKD 2	99(23.6%)	60(29.4%)
CKD 3	17(4.1%)	8(3.9%)
CKD 4	4(1.0%)	0
CKD 5	7(1.6%)	2(1.0%)
Pathologic stage		
pT1	322(76.8%)	161(78.9%)
pT2	46(11.0%)	25(12.3%)
pT3	45(10.7%)	15(7.4%)
pT4	6(1.5%)	3(1.4%)
Fuhrman grade		
1	132(31.5%)	68(33.3%)
2	177(42.2%)	87(42.6%)
3	95(22.7%)	45 (22.1%)
4	15(3.6%)	4(2.0%)
Histologic subtype		
Clear cell	359(85.7%)	186(91.2%)
Non-clear cell	60(14.3%)	18(9.8%)
Tumor necrosis		
Yes	17(4.1%)	4(2.0%)
No	402 (95.9%)	200(98.0%)
Tumor size, cm		
≥7	73(17.4%)	35(17.2%)
<7	346(82.6%)	169(82.8%)
Albumin, g/l		
≤35	48(11.5%)	29(14.2%)
>35	371(88.5%)	175(85.8%)
ALP, U/l		
≥125	21(5.0%)	11(5.4%)
<125	398(95.0%)	193(94.6%)
AAPR		
<0.39	54(12.9%)	25(12.3%)
≥0.39	365(87.1%)	179(87.7%)
Globulin, g/l		
≤25	75(17.9%)	41(20.1%)
>25	344(82.1%)	163(79.9%)
ALT, IU/l		
≥40	51(12.2%)	30(14.7%)
<40	368(87.8%)	174(85.3%)
AST, IU/l		
≥35	63(15.0%)	35(17.2%)
<35	356(85.0%)	169(82.8%)

Abbreviations: DM, diabetes mellitus; CKD stage, chronic kidney disease stage; ALP, alkaline phosphatase; AAPR, albumin-to-alkaline phosphatase; ALT, alanine aminotransaminase; AST, aspartate aminotransaminase.

**Table 2:** Associations between AAPR and clinicopathological characteristics in primary cohort.

Variables	AAPR<0.39 (n=54)	AAPR≥0.39 (n=365)	*P* value
Age, years			0.004*
>65	32(59.3%)	141(38.6%)	
≤65	22(40.7%)	224(61.4%)	
Gender			0.320
Male	31(57.4%)	130(35.6%)	
Female	23(42.6%)	235(64.4%)	
ASA grade			0.193
≥3	5(9.3%)	18(4.9%)	
<3	49(90.7%)	347(95.1%)	
BMI, kg/m^2^			0.087
≥25	7(13.0%)	85(23.3%)	
<25	47(87.0%)	280(76.7%)	
DM			0.160
Yes	23(42.6%)	120(32.9%)	
No	31(57.4%)	245(67.1%)	
Hypertension			0.091
Yes	28(51.9%)	145(39.7%)	
No	26(48.1%)	220(60.3%)	
Anemia			0.001*
Yes	17(31.5%)	48(13.2%)	
No	37(68.5%)	317(86.8%)	
Surgical approach			0.663
Partial nephrectomy	10(18.5%)	77(21.1%)	
Radical nephrectomy	44(81.5%)	288(78.9%)	
CKD stage			0.025*
CKD 1	37(68.5%)	255(69.9%)	
CKD 2	9(16.7%)	90(24.7%)	
CKD 3	3(5.6%)	14(3.8%)	
CKD 4	1(1.8%)	3(0.8%)	
CKD 5	4(7.4%)	3(0.8%)	
Pathologic stage			0.306
pT1	37(68.5%)	285(78.1%)	
pT2	6(11.1%)	40(11.0%)	
pT3	10(18.5%)	35(9.6%)	
pT4	1(1.9%)	5(1.3%)	
Fuhrman grade			0.531
1	17(31.5%)	115(31.5%)	
2	21(38.9%)	156(42.8%)	
3	12(22.2%)	83(22.7%)	
4	4(7.4%)	11(3.0%)	
Histologic subtype			0.911
Clear cell	46(85.2%)	313(85.8%)	
Non-clear cell	8(14.8%)	52(14.2%)	
Tumor necrosis			0.888
Yes	2(3.7%)	15(4.1%)	
No	52(96.3%)	350(95.9%)	
Tumor size, cm			0.820
≥7	10(18.5%)	63(17.3%)	
<7	44(81.5%)	302(82.7%)	
Globulin, g/l			0.163
≤25	6(11.1%)	69(18.9%)	
>25	48(88.9%)	296(81.1%)	
ALT, IU/l			0.004*
≥40	13(24.1%)	38(10.4%)	
<40	41(75.9%)	327(89.6%)	
AST, IU/l			0.016*
≥35	14(25.9%)	49(13.4%)	
<35	40(74.1%)	316(86.6%)	

*Statistically significant

**Table 3:** Multivariable regression analysis of clinicopathological parameters for the prediction of OS and CSS in primary cohort

Variables	OS		CSS	
	HR(95%CI)	*P* value	HR(95%CI)	*P* value
Age (>65 vs ≤65 years)	2.862(1.321-6.201)	0.008*	2.834(1.190-6.746)	0.019*
ASA grade (≥3 vs <3)	2.413(0.911-6.390)	0.076	1.623(0.481-5.479)	0.436
BMI (≥25 vs <25)	0.435(0.099-1.900)	0.268	-	-
Anemia (Yes vs No)	1.418(0.625-3.218)	0.404	1.464(0.566-3.788)	0.432
CKD stage				
CKD 1	1.000	1.000		
CKD 2-3 vs CKD 1	1.555(0.440-5.493)	0.493	-	-
CKD 4-5 vs CKD 1	3.052(0.744-12.508)	0.121		
Pathologic T stage				
pT1	1.000	1.000	1.000	1.000
pT2 vs pT1	1.771(0.557-5.633)	0.333	1.559(0.432-5.630)	0.498
pT3 vs pT1	3.153(1.227-8.101)	0.017*	4.258(1.487-12.192)	0.007*
pT4 vs pT1	32.425(6.683-157.335)	<0.001*	32.617(5.226-203.590)	<0.001*
Fuhrman grade (≥3 vs <3)	1.742(0.815-3.723)	0.152	2.403(0.996-5.798)	0.051
Tumor necrosis (Yes vs No)	2.687(0.885-8.159)	0.081	3.672(1.166-11.560)	0.026*
Tumor size (≥7 vs <7)	2.549(0.965-6.736)	0.059	2.619(0.973-7.054)	0.057
Albumin (<35 vs ≥35)	1.684(0.741-3.828)	0.213	1.417(0.520-3.861)	0.495
AAPR (<0.39 vs ≥0.39)	2.745(1.266-5.953)	0.011*	3.042(1.278-7.243)	0.012*

*Statistically significant

**Table 4:** Predictive ability comparison of models for OS and CSS with 1000 bootstraps

Model	c-index	95%CI
**Nomogram for OS**		
Model A = pT+Age+AAPR	0.821	0.750-0.892
Model B = pT+Age	0.772	0.690-0.854
**Nomogram for CSS**		
Model C = pT+Age+AAPR+Necrosis	0.839	0.756-0.922
Model D = pT+Age +Necrosis	0.809	0.723-0.895

**Table 5:** Predictive ability comparison of models for OS and CSS with ROC analysis

Model	Sensitivity (%)	Specificity (%)	Accuracy (%)	Youden index	Positive predictive value (%)	Negative predictive value (%)	Positive likelihood ratio	Negative likelihood ratio
**Nomogram for OS**								
Model A = pT+Age+AAPR	66.67	84.60	83.05	0.513	74.20	75.83	4.33	0.39
Model B = pT+Age	66.67	74.15	73.51	0.408	70.56	75.80	2.58	0.45
**Nomogram for CSS**								
Model C = pT+Age+AAPR+Necrosis	74.07	79.85	79.47	0.539	73.57	80.79	3.68	0.32
Model D = pT+Age +Necrosis	74.07	75.77	75.66	0.498	70.88	80.71	3.06	0.34

## Abbreviations

AAPR: albumin-to-alkaline phosphatase ratio
ALB: albumin
ALP: alkaline phosphatase
RCC: renal cell carcinoma
HCC: hepatocellular carcinoma
UTUC: upper tract urothelial carcinoma
OS: overall survival
MFS: metastasis-free survival
CSS: cancer-specific survival
CKD: chronic kidney disease
SSIGN: Stage, Size, Grade and Necrosis Score
UISS: UCLA Integrated Staging System
